# Ovulation and ovarian wound healing are impaired with advanced reproductive age

**DOI:** 10.18632/aging.103237

**Published:** 2020-05-14

**Authors:** Jamie N. Mara, Luhan T. Zhou, Megan Larmore, Brian Johnson, Rebecca Ayiku, Farners Amargant, Michele T. Pritchard, Francesca E. Duncan

**Affiliations:** 1 Department of Obstetrics and Gynecology, Feinberg School of Medicine, Northwestern University, Chicago, IL 60611, USA; 2 Department of Comparative Medicine, University of Washington, Seattle, WA 98195, USA; 3 Department of Pharmacology, Toxicology and Therapeutics, University of Kansas Medical Center, Kansas City, KS 66160, USA

**Keywords:** ovulation, fibrosis, wound healing, ovary, reproductive aging

## Abstract

Aging is associated with reduced tissue remodeling efficiency and increased fibrosis, characterized by excess collagen accumulation and altered matrix degradation. Ovulation, the process by which an egg is released from the ovary, is one of the most dynamic cycles of tissue wounding and repair. Because the ovary is one of the first organs to age, ovulation and ovarian wound healing is impaired with advanced reproductive age. To test this hypothesis, we induced superovulation in reproductively young and old mice and determined the numbers of eggs ovulated and corpora lutea (CLs), the progesterone producing glands formed post-ovulation. Reproductively old mice ovulated fewer eggs and had fewer CLs relative to young controls. Moreover, reproductively old mice exhibited a greater number of oocytes trapped within CLs and expanded cumulus oocyte complexes within unruptured antral follicles, indicative of failed ovulation. In addition, post-ovulatory tissue remodeling was compromised with age as evidenced by reduced CL vasculature, increased collagen, decreased hyaluronan, decreased cell proliferation and apoptosis, impaired wound healing capacity, and aberrant morphology of the ovarian surface epithelium (OSE). These findings demonstrate that ovulatory dysfunction is an additional mechanism underlying the age-related loss of fertility beyond the reduction of egg quantity and quality.

## INTRODUCTION

Ovulation, the process by which a mature egg is released from an antral follicle into the oviduct, is an essential biological process required for fertility and sustained cyclic endocrine function. During this process, there is degradation of the extracellular matrix (ECM) and complete loss of the ovarian surface epithelium (OSE) at the site of follicular rupture [1]. Following ovulation, the cells that remain in post-ovulatory follicles will undergo luteinization and form corpora lutea (CLs), the transient endocrine structures that produce progesterone [2]. If pregnancy does not occur, CLs will regress. In the mouse, ovulation occurs every estrous cycle (~4-5 days) and in the human, every menstrual cycle (~28 days) [3]. Female mice and women will undergo approximately 650 and 400 ovulations over the course of their reproductive lifespans, respectively [4]. Thus, ovulation is one of the most dynamic examples of physiological wound repair in biology.

Ovulation is essential for fertility because ovarian follicles in progesterone receptor (PR) knockout mice develop normally to the preovulatory stage but fail to ovulate due to a lack of two PR-dependent proteases, ADAMTS-1 and cathepsin L, rendering these animals infertile despite normal follicular development [5, 6]. Additionally, ovulation is important for optimal endocrine function. In the rodent estrous cycle and human menstrual cycle, estrogen slowly rises until the luteinizing hormone (LH) surge occurs to induce ovulation. The CLs that form will then secrete progesterone, which will become the dominant hormone until CL regression in mice or the formation of a corpus albicans in humans, and the renewal of the estrous or menstrual cycle, respectively [7]. Thus, the continuous cycle of folliculogenesis, ovulation, and CL formation and regression are essential for fertility and endocrine function and require the ovary to be an exceptionally dynamic organ.

Tissue dysfunction occurs with age, and several organ systems experience age-related dysregulated ECM remodeling and wound healing. One example is the skin which, as the barrier to the external environment, must be consistently renewed for protection and tissue homeostasis. When the epidermis of young and old mice is wounded, the skin of young mice repairs faster and more effectively than old [8]. The gastrointestinal epithelium is also another tissue that undergoes rapid turnover. In aged rats, disrupted remodeling of tight junctions gives rise to an altered epithelial barrier and geriatric intestinal dysfunction [9]. Additionally, multiple organ systems become fibrotic with age including the lung [10], heart [11], and kidney [12]. Fibrosis is characterized by excess ECM deposition, particularly collagen I and III, and can result in and contribute to dysregulated tissue remodeling and wound healing [13, 14].

We and others have demonstrated that the ovarian stroma becomes fibrotic with age [15, 16]. However, the functional consequences of this fibrotic transition in the ovary have not been investigated. We hypothesized that with advanced reproductive age and increased fibrosis, ovulation and ovarian wound healing is impaired. To test this hypothesis, we hyperstimulated and induced ovulation in reproductively young and old mice and compared the number of eggs ovulated to the number of CLs formed in the ovarian tissue. Reproductively old mice exhibited a greater number of oocytes trapped within CLs and expanded cumulus oocyte complexes within unruptured antral follicles, phenotypes consistent with failed ovulation. To examine ovarian tissue remodeling post-ovulation, we examined multiple parameters, including the ovarian ECM, cell proliferation and death, and properties of the OSE. Post-ovulation, ovaries from reproductively old mice contained more collagen and less hyaluronan and exhibited less cell proliferation and apoptosis. Additionally, wound repair efficiency was compromised in ovaries from reproductively old mice both *in vivo* and *in vitro*, and the OSE had aberrant age-associated morphologies including increased thickness, number of invaginations into the stroma, and number of epithelial outgrowths. These findings demonstrate that reproductive age is associated with ovulation and wound healing dysfunction which may provide an explanation for why fewer eggs are ovulated in women of advanced reproductive age beyond a loss of ovarian reserve. These findings may also implicate ovulatory dysfunction in other conditions where the ovary is associated with increased stiffness or fibrosis such as polycystic ovarian syndrome (PCOS) and iatrogenic infertility following treatment with chemotherapy or radiation [17–20].

## RESULTS

### Reproductively young and old mice have a similar hormone response to hyperstimulation

**Figure 1:**
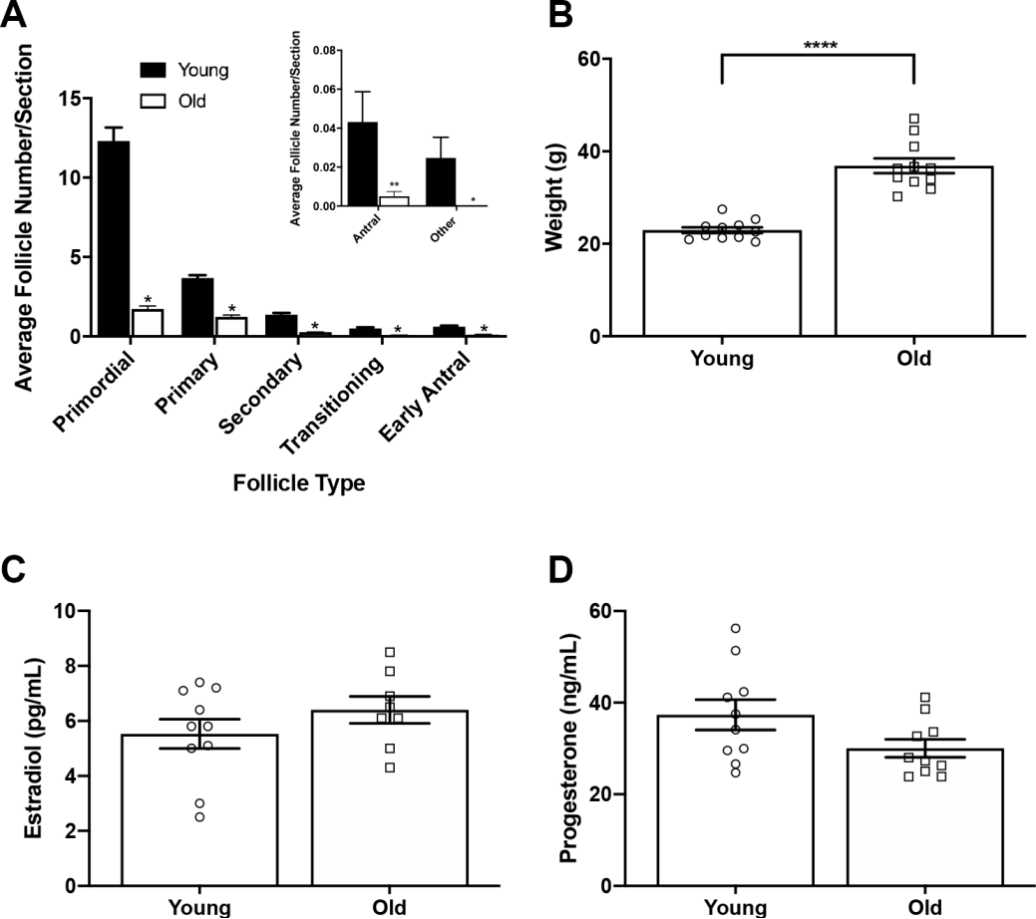
**Reproductively old mice weigh more than young mice but respond similarly to hormone stimulation.** (**A**) Graph showing the average number of each follicle class per ovarian section (every fifth section of serially sectioned ovaries was counted; N = 20 ovaries from 5 reproductively young and 5 old mice). The inset shows the average number of antral follicles and other (degenerate) follicles per ovarian section with a different scale. T-tests were performed within each follicle class; asterisks denote P < 0.0001. (**B**) Weights of reproductively young and old mice (n = 11 young and 11 old mice). A t-test was performed; asterisks denote P < 0.0001. (**C**) Serum estradiol levels were assessed (reportable range 3-300 pg/mL). A t-test was performed and there was no difference in estradiol levels between age cohorts. (**D**) Serum progesterone levels were assessed (reportable range 0.15-40 ng/mL). A t-test was performed and there was no difference between age cohorts. Data are represented as mean **±** SEM. N = 20 samples for hormone analysis.

To examine if ovulation and remodeling are affected by age, we first confirmed our physiologic aging model. The mice we used to represent advanced reproductive age were 14-17 months old, which based on a linear extrapolation estimate, corresponds to women 38-45 years old [21]. Furthermore, this specific mouse strain demonstrates many reproductive aging phenotypes including reduced ovarian reserve, increased egg aneuploidy, ovarian stromal fibrosis, and altered adiposity [15, 21–23]. We confirmed this model by performing follicle counts in mice following hyperstimulation and superovulation since reduced follicle numbers are a hallmark of reproductive aging [24]. We observed a significant reduction in the number of follicles in all classes in old mice relative to young, consistent with what has been observed previously in unstimulated mice of the same strain [23] (Figure 1A). We noted significant weight differences between age cohorts, with reproductively young mice weighing 23.0 ± 2.13 g and old mice weighing 36.9 ± 5.26 g (P < 0.0001; Figure 1B). Due to this difference in weight, we wanted to validate that both cohorts of mice responded similarly to hyperstimulation. Thus, serum estradiol and progesterone levels were measured for each animal (Figure 1C, 1D). Serum estradiol was similar between reproductively young and old mice; 5.5 ± 0.53 pg/mL and 6.4 ± 0.49 pg/mL, respectively (P > 0.05; Figure 1C). Serum progesterone was also similar between age cohorts; 37.4 ± 3.32 ng/mL in reproductively young mice and 30.1 ± 1.96 ng/mL in old mice (P > 0.05; Figure 1D). These results indicate that a similar hormone response was achieved in both age cohorts despite weight differences.

### Reproductively old mice ovulate fewer eggs and have fewer CLs relative to young mice

**Figure 2:**
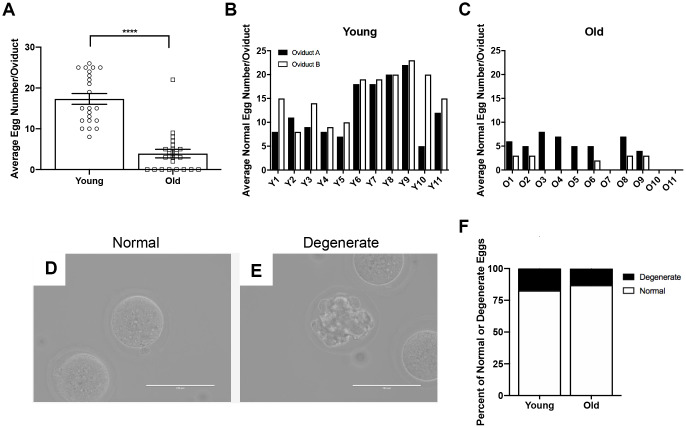
**Reproductively old mice ovulate fewer eggs than reproductively young mice, but the proportion of normal and degenerate eggs does not differ between age groups.** (**A**) The number of eggs ovulated into the oviducts of each mouse was tracked, and the graph shows the average number of eggs ovulated per mouse (N = 11 mice per age group, 22 oviducts). A t-test was performed; asterisks denote P < 0.0001. Data are represented as mean **±** SEM. The number of morphologically normal eggs ovulated and retrieved from each oviduct from (**B**) reproductively young and (**C**) old individual mice. Representative brightfield microscopy images of morphologically (**D**) normal and (**E**) degenerate eggs. Scale bars are 100 μm. (**F**) The graph shows the proportion of morphologically normal and degenerate eggs ovulated from both age cohorts.

**Figure 3:**
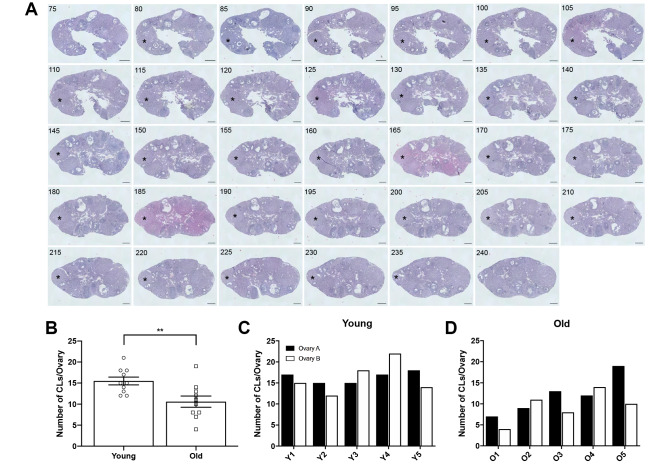
**Ovaries from reproductively old mice contain fewer CLs than young mice.** (**A**) Representative H&E-stained ovarian tissue sections demonstrate the CL tracking process. Numbers in the upper left corner indicate the tissue section number. Black asterisks identify the same CL that appears across tissue sections. Indicated CL was visible beginning in section 75 and ending in section 240. Scale bars are 100 μm. (**B**) Graph showing the number of CLs per ovary for both age cohorts. A t-test was performed; asterisks denote significance (P = 0.008) (N = 5 mice, 10 ovaries per age group). Data are represented as mean **±** SEM. The number of CLs per ovary for all (**C**) reproductively young and (**D**) reproductively old mice.

To determine if the number of eggs ovulated differed between reproductively young and old mice, we collected the eggs from the ampullae of all mice. Fewer eggs per oviduct from reproductively old mice were recovered compared to young controls; 4.5 ± 1.16 eggs per oviduct and 17.3 ± 1.32 eggs, respectively (P < 0.0001; Figure 2A, Table 1). In addition to examining all animals as an aggregate, we examined the number of eggs ovulated from individual animals. Overall, reproductively young mice tended to ovulate between 10 and 20 eggs per oviduct while reproductively old mice tended to ovulate fewer than 10, with no eggs recovered from some animals (Figure 2B, 2C). We also assessed gamete morphology by transmitted light microscopy and there were no age-dependent differences in the proportion of morphologically normal and degenerate eggs ovulated in reproductively young and old mice (P > 0.05; Figure 2D–2F, Table 1). Each egg ovulated should produce a single CL. Therefore, through analysis of serial histological sections, we determined the total number of CLs in ovarian tissue as an aggregate and from each individual animal across age cohorts (Figure 3A–3D, Table 1). Similar to the number of eggs, there were fewer CLs in reproductively old animals relative to young; 10.6 ± 1.34 CLs per ovary and 15.5 ± 0.93 CLs per ovary, respectively (P = 0.008; Figure 3B). Individual reproductively young mice tended to have similar numbers of CLs to each other while there was more variation in numbers among reproductively old mice (Figure 3C, 3D).

**Table 1:** Summary of ovulation results by animal used for CL analysis.

	**Mouse**	**Side**	**Total eggs ovulated (#)**	**Normal eggs ovulated (#)**	**Degenerate cells ovulated (#)**	**CL (#)**
**Young**	**Y1**	A	10	8	2	17
		B	15	15	0	15
	**Y2**	A	12	11	1	15
		B	10	8	2	12
	**Y3**	A	13	9	4	15
		B	14	14	0	18
	**Y4**	A	8	8	0	17
		B	10	9	1	22
	**Y5**	A	12	7	5	18
		B	10	10	0	14
**Old**	**O1**	A	6	6	0	7
		B	3	3	0	4
	**O2**	A	5	5	0	9
		B	3	3	0	11
	**O3**	A	8	8	0	13
		B	0	0	0	8
	**O4**	A	7	7	0	12
		B	0	0	0	14
	**O5**	A	0	0	0	19
		B	5	5	0	10

**Figure 4:**
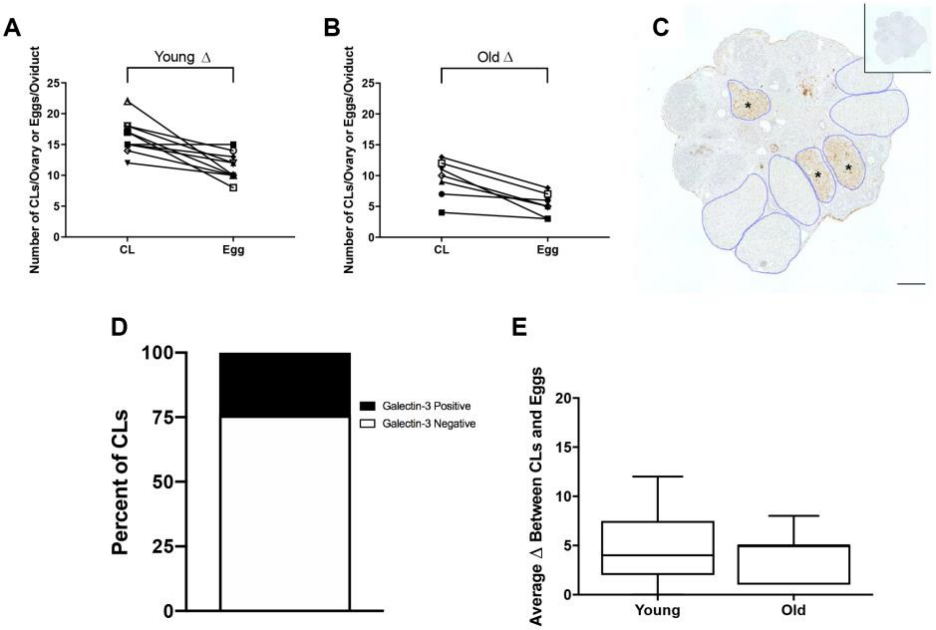
**The discrepancy between eggs ovulated and CLs tracked is partially accounted for by regressing CLs from previous estrous cycles.** (**A**) The number of CLs tracked per ovary and the number of eggs ovulated per oviduct for reproductively young mice (N = 5 mice, 10 ovaries). (**B**) The number of CLs tracked per ovary and the number of eggs ovulated per oviduct for reproductively old mice (N = 5 mice, 10 ovaries). (**C**) Representative image of Galectin-3 IHC in an ovary from a reproductively young mouse. All CLs are outlined in blue. Galectin-3 positive CLs are indicated with black asterisks. The inset shows the IgG control. The scale bar is 100 μm. (**D**) Graph showing the percent of Galectin-3 positive CLs (~24.3%) and Galectin-3 negative CLs (~75.7%) (N = 20 ovaries, 3 slides per ovary). (**E**) The graph shows the average discrepancy between CLs and eggs for both age cohorts. A t-test was performed and there was no difference in the discrepancy between the age cohorts (P > 0.05).

Although each ovulated egg should correspond to one CL in ovarian tissue, we observed more CLs in histological sections of both reproductively young and old mice compared to the number of eggs ovulated in the corresponding oviduct (Figure 4A, 4B). In mice, complete regression of CLs takes multiple estrous cycles, so the ovaries of mature mice can contain CLs from up to three different estrous cycles [25]. To determine the proportion of regressing CLs from previous estrous cycles to fresh CLs from the current hyperstimulation and superovulation protocol, we performed immunohistochemistry (IHC) against Galectin-3, a protein that binds to infiltrating macrophages and beta 1 integrin in the bovine CL [26] and whose expression in mice is synchronized with the synthesis of 20α-hydroxysteroid dehydrogenase (HSD), an enzyme expressed in regressing CLs [27]. Thus, Galectin-3 staining marks old CLs from previous cycles. Based on the number of Galectin-3 positive CLs, we determined that approximately a quarter of CLs in the ovaries we examined were from previous estrous cycles, which could account in part for the discrepancy between the number of eggs ovulated and CLs tracked in each animal (Figure 4C, 4D). The discrepancy between egg and CL number was similar between reproductively young and old mice; 4.9 ± 1.15 and 4.1 ± 0.94, respectively. (P > 0.05; Figure 4E). These results suggest that reproductively old mice ovulate fewer eggs and have fewer CLs than young mice, but the proportion of eggs ovulated to CLs is similar between age cohorts.

### Reproductively old mice exhibit a greater percentage of failed ovulations compared to young mice

**Figure 5:**
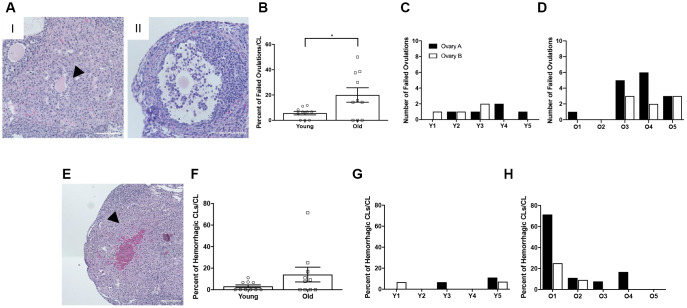
**Reproductively old mice exhibit more failed ovulation events.** (**A**) Representative H&E-stained ovarian tissue sections depicting two phenotypes indicative of failed ovulation events including (I) trapped oocytes within CLs and (II) expanded unruptured antral follicles. Scale bars are 100 μm. (**B**) Graph showing the percent of failed ovulations per CL number for reproductively young and old mice (N = 10 ovaries per age group). A t-test was performed and P = 0.025. Data are represented as mean **±** SEM. The number of failed ovulations per ovary for all (**C**) reproductively young and (**D**) reproductively old mice. (**E**) Representative H&E-stained ovarian tissue section depicting a hemorrhagic CL. The scale bar is 50 μm. (**F**) Graph showing the percent of hemorrhagic CLs for both age cohorts (N = 10 ovaries per age group). A t-test was performed and there was no significant difference (P > 0.05). Data are represented as mean **±** SEM. The percent of hemorrhagic CLs for all (**G**) reproductively young and (**H**) reproductively old mice.

To investigate whether there was additional evidence of ovulation dysfunction in reproductively old mice, we examined two phenotypes of ovulatory failure. The first of these phenotypes was oocytes trapped within CLs, or luteinized but unruptured follicles. The presence of an oocyte in a CL indicates that the follicle luteinized before rupture and the oocyte was not ovulated [28] (Figure 5AI). This could be due to follicles that luteinized prior to rupture or luteinization that occurred because follicles failed to rupture. The other phenotype was expanded cumulus oocyte complexes within large antral follicles (Figure 5AII). Following the LH surge, cumulus cells surrounding the oocyte undergo expansion prior to follicular rupture. Cumulus cell expansion indicates follicular response to hormonal stimulation, but if the oocyte is still present within the antral follicle, ovulation clearly failed to occur [29]. Reproductively old mice had a higher percentage of failed ovulatory events compared to young controls; 20.0 ± 5.71% and 5.7 ± 1.39%, respectively (P = 0.025; Figure 5B). Of note, failed ovulations were observed across individual reproductively young mice, and even though some ovaries from reproductively old mice did not show evidence of failed ovulations, the prevalence tended to be greater when examined across individual animals (Figure 5C, 5D). Hemorrhagic CLs, characterized by large areas infiltrated with red blood cells, were also observed in some CLs, likely indicative of very recent ovulation (Figure 5E). However, there was no difference in the prevalence of this phenotype between reproductively young and old mice when examined in aggregate or across individual animals (Figure 5F–5H).

Although 5 IU PMSG and hCG are routinely used for hyperstimulation and superovulation in mice [30], this supraphysiologic hormone dose may drive the system and obscure more subtle age-associated phenotypes. Therefore, we performed the same histological analyses on ovaries from reproductively young and old mice treated with 2.5 IU PMSG and hCG, half of the original dose. Even with the half dose paradigm, similar estrogen and progesterone responses were achieved in both age cohorts (Supplementary Figure 1A–1C). Furthermore, similar trends to what occurred with the full dose of hormones were observed. Reproductively old mice ovulated fewer eggs and had fewer CLs than young mice, and there was a similar age-associated increase in the number failed ovulations, although this was not statistically significant (Supplementary Figure 1D–1F).

### CLs from reproductively old mice are structurally different than CLs from young mice

**Figure 6:**
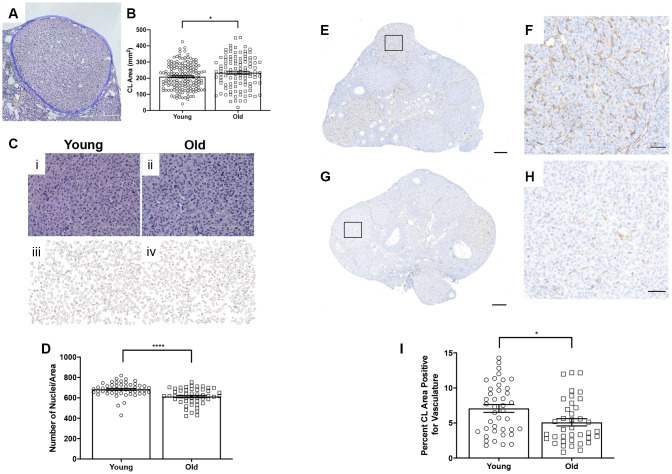
**CLs exhibit age-associated structural differences.** (**A**) Representative H&E-stained CL. The perimeter of the CL is outlined in blue. The scale bar is 50 μm. (**B**) Graph showing the area of each CL. Each data point corresponds to one CL. A t-test was performed; asterisk indicates significance (P = 0.022). (**C**) Images showing how luteinized area was determined at 40X magnification and the same images in ImageJ software. (**D**) Graph showing the average number of nuclei per defined CL area. Each data point corresponds to one CL. A t-test was performed; asterisks denote P < 0.0001. (**E**) Representative image of CD31 staining in reproductively young ovarian tissue with the boxed region in (**F**) and (**G**) representative image of CD31 staining in reproductively old ovarian tissue with the boxed region in (**H**). (**I**) Graph showing the percent of CL area positive for vasculature. A t-test was performed (P = 0.01). Scale bars are (**E**, **G**) 200 μm and (**F**, **H**) 50 μm. Data are represented as mean **±** SEM.

To examine whether there were any age-related structural differences in the CLs that were produced, we analyzed the number of histological sections a CL spanned as a surrogate marker of volume and measured the largest cross-sectional area of each CL. Although there was no difference in the number of sections CLs spanned between age groups (data not shown), CLs from reproductively old mice had a larger average cross-sectional area than CLs from young controls; 234.1 ± 9.48 mm^2^ and 209.3 ± 6.08 mm^2^, respectively (P = 0.028; Figure 6A–6C). Following ovulation, the granulosa and theca cells luteinize and form the CL. A hallmark of this transition is cytoplasmic hypertrophy, which can be quantified as an increased cytoplasmic: nuclear ratio. To determine if the degree of hypertrophy was different between reproductively young and old mice, we counted the number of nuclei per defined area of CLs (Figure 6D). This value is an indirect measure of hypertrophy, as fewer nuclei per area indicate more cytoplasm [31]. CLs from reproductively old mice had fewer nuclei per area than young; 612.5 ± 11.55 and 683.4 ± 9.53, respectively (P < 0.0001; Figure 6E).

CLs are highly vascularized structures. The extensive vascular network in CLs provides the cells with the oxygen and nutrients necessary to produce and subsequently secrete progesterone [32]. To determine if CL vasculature differed with age, we performed IHC using an endothelial cell marker, CD31 (Figure 6E–6H). The percentage of CD31 positive area of CLs was significantly higher in CLs from reproductively young ovarian tissue relative to old; 7.1 ± 0.56% and 5.1 ± 0.52%, respectively (P = 0.01; Figure 6I). Thus, CLs are less vascular with advanced reproductive age.

### Remodeling of the ovarian matrix post-ovulation is altered with advanced reproductive age

**Figure 7:**
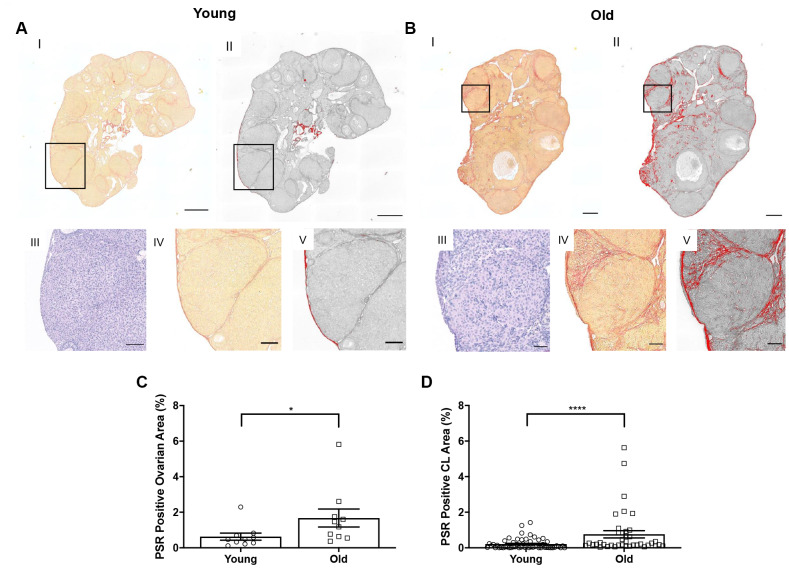
**Collagen significantly increases in the whole ovary and around CLs in reproductively old mice.** (**A**) Representative images depicting (I) PSR staining in a reproductively young ovary and (II) the same image in ImageJ software. (III) Representative image of an H&E-stained CL, (IV) the same CL with PSR staining, and (V) the CL in ImageJ. Scale bars are (I-II) 100 μm and (III-V) 25 μm. (**B**) Representative images of a reproductively old ovary and CL with the same histological techniques in (**A**). Scale bars are (I-II) 100 μm and (III-V) 25 μm. (**C**) Graph showing the percent of reproductively young and old ovaries positive for PSR (N = 10 ovaries per age group). A t-test was performed; asterisk denotes P = 0.019. (**D**) Graph showing analysis of the percent of PSR positive area in and surrounding CLs for both age cohorts. Each data point corresponds to one CL. A t-test was performed; asterisks denote P < 0.0001. Data are represented as mean **±** SEM (**C** and **D**).

Although we have previously demonstrated an increase in stromal collagen with advanced reproductive age in unstimulated mice [15], we wanted to examine collagen in stimulated mice post-ovulation. To visualize collagen, we stained ovarian tissue sections from reproductively young and old mice with Picrosirius Red (PSR), a validated histological stain specific to collagen I and III [33] (Figure 7A, 7B). The percentage of ovarian area positive for PSR staining was higher in reproductively old mice compared to young; 1.7 ± 0.51% and 0.6 ± 0.20%, respectively (P = 0.019; Figure 7C). We also examined whether there was an enrichment of collagen immediately surrounding CLs from reproductively old mice, which may be indicative of compromised post-ovulatory ECM remodeling. Consistent with the whole ovary, a higher proportion of the area within and surrounding CLs was PSR positive in reproductively old ovaries relative to young; 0.8 ± 0.20% and 0.2 ± 0.04%, respectively (P < 0.0001; Figure 7D).

**Figure 8:**
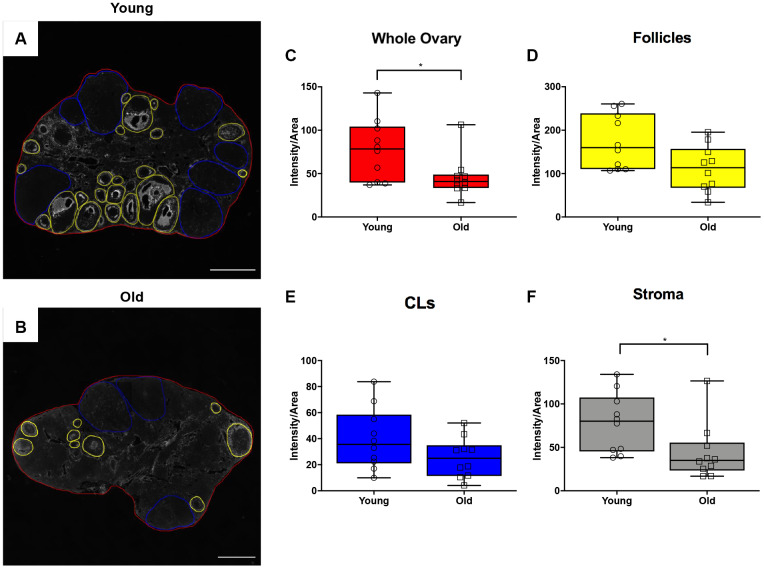
**Hyaluronan significantly declines in the ovarian stroma of reproductively old mice.** (**A**) Representative image of the hyaluronan binding protein (HABP) assay performed with reproductively young ovaries. The whole ovarian area is outlined in red. Follicles are outlined in yellow. CLs are outlined in blue. (**B**) Representative image of the HABP assay performed on reproductively old ovaries with the same ovarian sub-compartments outlined. Scale bars are (**A**, **B**) 200 μm. Graphs showing hyaluronan as intensity per area within (**C**) whole ovaries, (**D**) follicles, (**E**) CLs, and (**F**) the ovarian stroma between age cohorts. T-tests were performed; asterisks indicate significant differences (C: P = 0.043; F: P = 0.012). Data are represented as mean **±** SEM (**C**–**F**). N = 10 ovaries per age group.

In addition to collagen, another prominent component of the ovarian ECM is hyaluronan (HA), which promotes tissue homeostasis and hydration and is involved in wound healing [34–37]. Because HA is non-immunogenic, antibody-based approaches cannot be used for its localization and quantification. Therefore, we performed a hyaluronan binding protein (HABP) assay that makes use of specific proteins that recognize and bind to HA [38, 39] on ovarian tissue sections from reproductively young and old mice and analyzed the fluorescent signal in a sub-compartment specific manner (Figure 8A, 8B). This analysis allowed us to determine hyaluronan content in the whole ovary and in the follicles, CLs, and the ovarian stroma after superovulation. Hyaluronan was significantly lower in ovaries from reproductively old mice relative to young counterparts (P = 0.043; Figure 8C). Although the hyaluronan content in follicles and CLs tended to decline in aged ovaries, this decrease was only significant in the ovarian stromal compartment (P = 0.012; Figure 8D–8F). These data suggest that advanced reproductive age is associated with alterations in remodeling of both the collagen and hyaluronan matrices post-ovulation.

### Reproductively old ovaries undergo less proliferation and cell death post-ovulation suggesting altered general tissue remodeling

**Figure 9:**
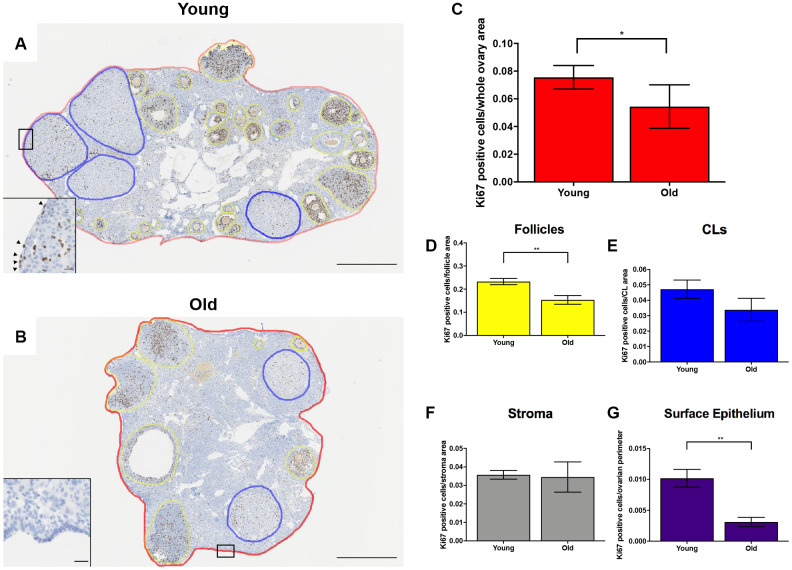
**Cell proliferation is reduced post-ovulation in reproductively old mice.** (**A**) Representative image of IHC labeling Ki67 in reproductively young ovaries. The whole ovarian area is outlined in red. Follicles are outlined in yellow. CLs are outlined in blue. (**B**) Representative image of IHC labeling Ki67 in reproductively old ovaries with the same ovarian sub-compartments outlined. Insets depict the boxed region of the OSE in each respective image. Scale bars are (**A**, **B**) 500 μm and (insets) 25 μm. Graphs showing the number of Ki67 positive cells per sub-compartment are within (**C**) whole ovaries, (**D**) follicles, (**E**) CLs, (**F**) the ovarian stroma, and (**G**) the OSE. T-tests were performed; asterisks indicate significant differences (**C**: P = 0.0294; **D**: P = 0.0091; **G**: P = 0.0023). Data are represented as mean **±** SEM (**C**–**G**). N = 5 ovaries per age group.

Given the altered status of the ovarian matrix post-ovulation, we examined cell proliferation and death as the balance between these two processes is essential for optimal tissue remodeling. Cell proliferation contributes to tissue growth while cell death maintains homeostasis by removing unwanted or damaged cells [40]. To investigate cell proliferation post-ovulation with reproductive age, we performed IHC to localize Ki67 within ovarian tissue from both age cohorts (Figure 9A, 9B). Ki67 is a well-established marker of active cell proliferation as it is a nuclear antigen present during all active phases of the cell cycle but not in quiescent cells [41, 42]. There were fewer Ki67 positive cells in the ovaries of reproductively old mice relative to young; 0.054 ± 0.007 and 0.076 ± 0.004 cells per area, respectively (P = 0.029; Figure 9C). There was a decline in the number of Ki67 positive cells in the follicular compartment between young and old mice; 0.23 ± 0.013 vs. 0.15 ± 0.019 Ki67 positive cells per follicle area, respectively (P = 0.009; Figure 9D). Similar numbers of Ki67 positive cells were observed in the CL and the stroma between age cohorts, although there was a downward trend in the CL (P > 0.05; Figure 9E, 9F). We also investigated OSE cell proliferation because the OSE is the main site of follicular rupture during ovulation and must undergo significant repair. There were significantly more Ki67 positive cells in the OSE of reproductively young mice relative to old; 0.01 ± 0.001 and 0.003 ± 0.0007 positive cells/ovarian perimeter, respectively (P = 0.002; Figure 9G).

**Figure 10:**
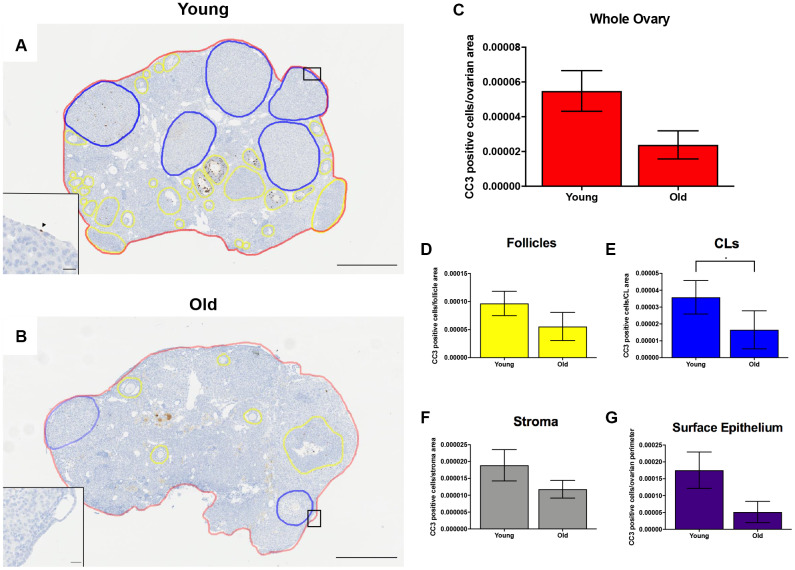
**Cell death is reduced post-ovulation in reproductively old mice.** (**A**) Representative image of IHC labeling CC3 in reproductively young ovaries. The whole ovarian area is outlined in red. Follicles are outlined in yellow. CLs are outlined in blue. (**B**) Representative image of the IHC labeling CC3 in reproductively old ovaries with the same ovarian sub-compartments outlined. Insets depict the boxed region of the OSE in each respective image. Scale bars are (**A**, **B**) 500 μm and (insets) 25 μm. Graphs showing the number of CC3 positive cells per sub-compartment are within (**C**) whole ovaries, (**D**) follicles, (**E**) CLs, (**F**) the ovarian stroma, and (**G**) the OSE. T-tests were performed; asterisks indicate significant differences (**E**: P = 0.022). Data are represented as mean **±** SEM (**C**–**G**). N = 5 ovaries per age group.

To investigate cell death in the ovary post-ovulation, we performed IHC to localize cleaved caspase 3 (CC3), a well-established marker of apoptosis which is responsible for the majority of proteolytic cleavage during apoptosis (Figure 10A–10B) [43]. There was a consistent trend of decreased CC3 positive cells with advanced age across ovarian area and sub-compartments, including the follicles, ovarian stroma, and OSE. However, this difference was only significant in the CLs (P = 0.022; Figure 10C–10G). Collectively, these data suggest reduced cell proliferation and apoptosis during post-ovulatory remodeling with advanced reproductive age. Because an optimal balance between proliferation and apoptosis is necessary for proper tissue remodeling, the decline in these processes with advanced reproductive age is indicative of dysregulated tissue repair.

### The OSE does not reform as effectively after wounding in vivo and in vitro with advanced reproductive age

**Figure 11:**
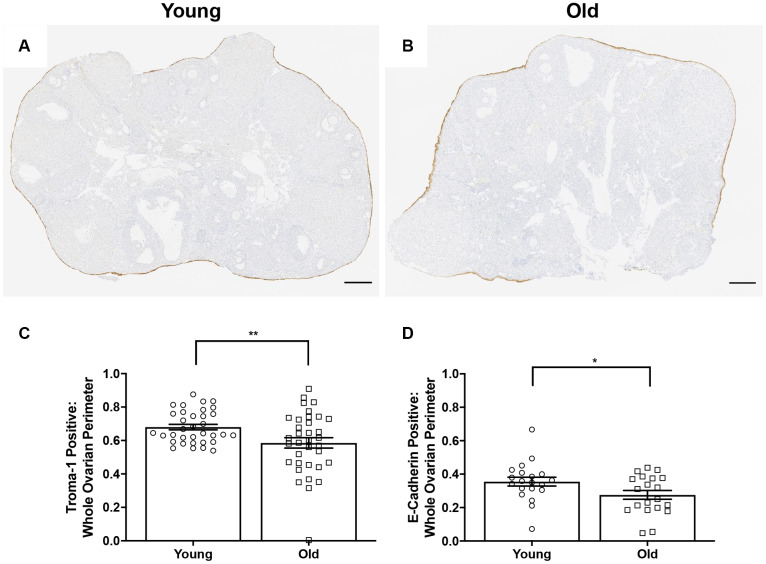
**OSE reformation post-ovulation *in vivo* is reduced with age.** Representative image of Troma-1 IHC in (**A**) reproductively young ovaries and (**B**) reproductively old ovaries. Scale bars are 200 μm (**A**, **B**). Graphs showing the proportion of the ovarian surface that was (**C**) Troma-1 positive and (**D**) E-Cadherin positive. T-tests were performed for both parameters and asterisks denote significance (**C**: P = 0.01; **D**: P = 0.04). For Troma-1, N = 10 ovaries per age group, each ovary was analyzed in triplicate or quadruplicate (N = 34 measurements for reproductively young ovaries, N = 35 measurements for reproductively old ovaries). For E-Cadherin, N = 10 ovaries per age group (N = 20 measurements for reproductively young ovaries, N = 20 measurements for reproductively old ovaries).

Because the OSE is a key structure that must be repaired following ovulation, we further investigated the ability of the OSE to repair itself with age. We performed IHC on ovarian sections with an antibody recognizing the epithelial cell marker, cytokeratin-8 (Troma-1) in ovaries from young and old mice (Figure 11A–11B) [44–46]. We determined the proportion of the ovarian surface that was Troma-1 positive relative to the entire ovarian perimeter to assess reformation and continuity of the OSE post-ovulation. A lower proportion of each ovary was lined by Troma-1 positive cells in reproductively old compared to young mice; 0.59 ± 0.031 and 0.68 ± 0.017, respectively (P = 0.01; Figure 11C). We performed the same analysis using an antibody recognizing E-Cadherin, another epithelial cell marker that induces cells in remodeling tissue to stop migrating and begin wound healing as part of the epithelial-mesenchymal transition (EMT) [47] (Figure 11D). As observed with Troma-1, ovaries from reproductively old mice had a smaller proportion of E-Cadherin positive cells relative to the entire ovarian surface compared to young controls; 0.28 ± 0.026 and 0.35 ± 0.026, respectively (P = 0.04). We further analyzed these results to determine the proportion of CL surface which was Troma-1 and E-Cadherin positive relative to the entire surface of all CLs to determine if remodeling deficits were specific to the CLs. We also analyzed the proportion of the remaining ovarian surface that was Troma-1 and E-Cadherin positive. As observed with the general remodeling findings, the proportion of CL surface and remaining ovarian surface positive for these two epithelial markers tended to be lower in reproductively old mice (Supplementary Figure 2). There was significantly less E-Cadherin positive staining in reproductively old mice, which suggests that impaired remodeling is specific to CLs (P = 0.0079). These results are consistent with age-associated defects in post-ovulatory wound repair.

**Figure 12:**
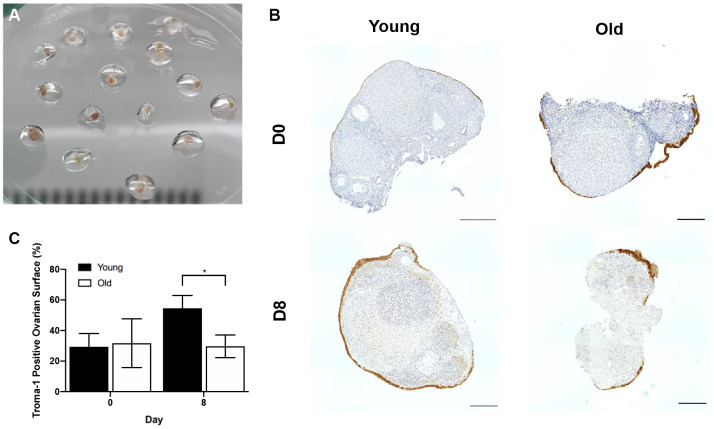
**The *in vitro* wound healing ability of the OSE is compromised with age.** (**A**) Ovary pieces encapsulated in alginate hydrogel beads. (**B**) Representative images of Troma-1 IHC with reproductively young (n = 7) and old ovary pieces (n = 7) at Day 0 and Day 8 of culture. Ovaries were fixed at each timepoint, therefore, the ovaries are shown at D0 and D8 are not the same. Scale bars are 100 μm. (**C**) Graph showing the average percent area of ovarian pieces encapsulated by Troma-1 positive cells. T-tests were performed; asterisk denotes significance (P = 0.029). Data are represented as mean **±** SEM.

To examine if age-related differences in OSE dynamics post-ovulation were intrinsic to the remodeling capacity of ovarian tissue, we used an *in vitro* wound healing assay. In this method, ovaries from reproductively young and old mice were wounded by cutting them into pieces followed by encapsulation and culture in alginate hydrogels for 8 days (Figure 12A). Previous studies indicate that the OSE will reform in a time-dependent manner following wounding in this system, and the degree to which this occurs can be quantified as the percent of each tissue piece surrounded by OSE over time [44, 45, 48]. At Day 0 of culture, there was no difference in the percent area of the ovarian tissue surface covered by epithelium between young and old mice; 29.4 ± 8.61% and 31.7 ± 15.92%, respectively (P > 0.05; Figure 12B, 12C). However, by Day 8, the reformation of the ovarian surface was greater in reproductively young mice relative to old; 54.5 ± 8.38% and 29.7 ± 7.43%, respectively (P = 0.029; Figure 12B, 12C). These data recapitulate our *in vivo* observations and demonstrate that impaired ovarian surface reformation is an inherent property of aged ovaries.

### The OSE exhibits age-associated changes in morphology

**Figure 13:**
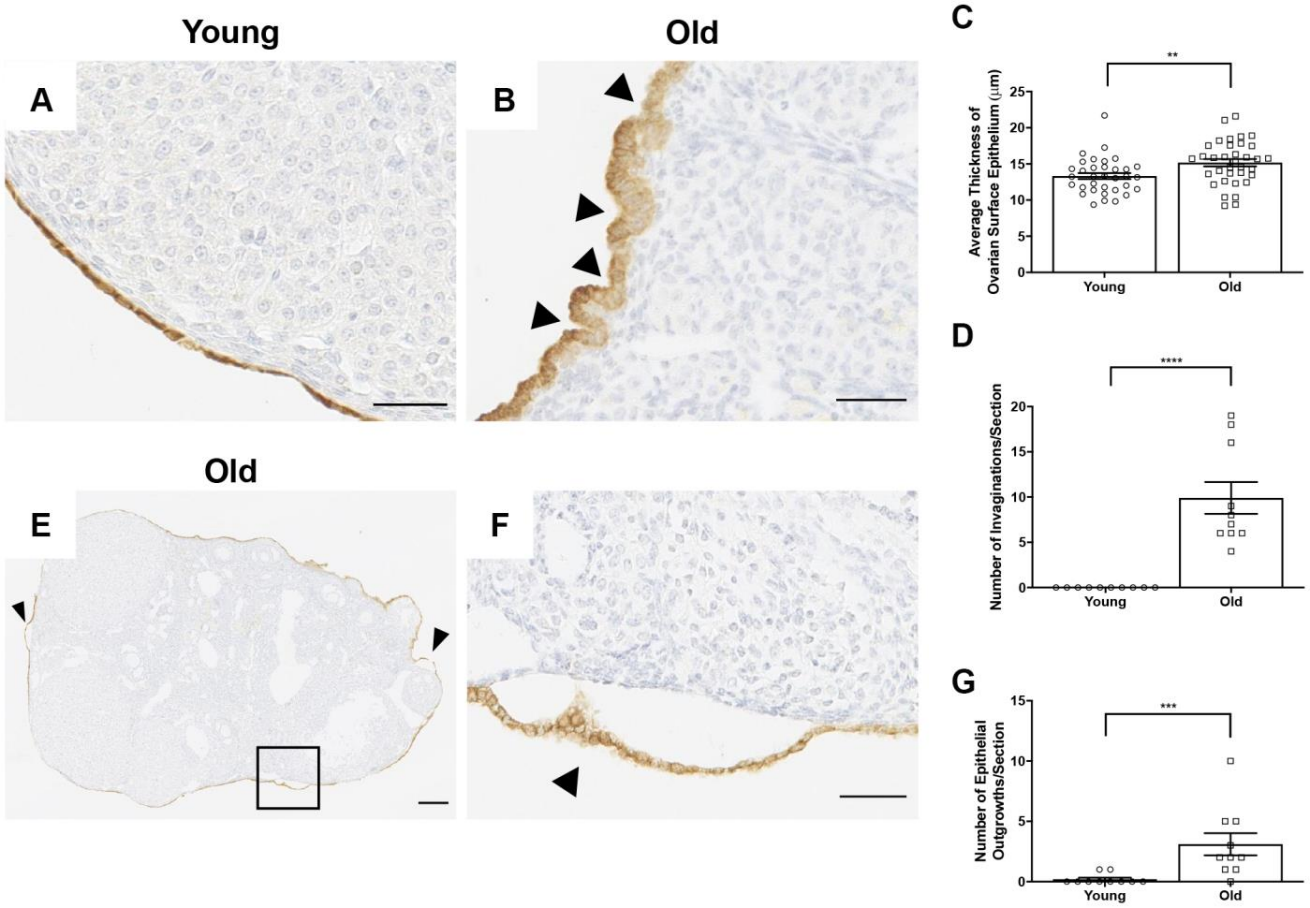
**The OSE exhibits aberrant morphologies in reproductively old ovaries.** (**A**) Representative 40X image of the OSE in an ovary from a reproductively young mouse. (**B**) Representative 40X image of the OSE in an ovary from a reproductively old mouse. Arrowheads indicate invaginations of the OSE into the ovarian stroma. (**C**) Graph showing the average thickness of the OSE for all reproductively young and old mice. N = 10 ovaries per age group, each ovary was analyzed in triplicate or quadruplicate (N = 34 measurements for reproductively young ovaries, N = 35 measurements for reproductively old ovaries). (**D**) The graph shows the number of invaginations per ovary section for all mice. N = 10 ovaries per age group. (**E**) Representative image of an aged ovary. Arrowheads indicate epithelial growths. (**F**) High magnification region of the boxed region in (**E**) depicting an epithelial outgrowth. (**G**) The graph shows the mean number of epithelial outgrowths per ovary section for all mice. N = 10 ovaries per age group. T-tests were performed on all measured parameters with asterisks indicating significant differences (**C**: P = 0.007; **D**: P < 0.0001; **E**: P = 0.0007). Scale bars are (**A**, **B**, **F**) 50 μm and (**E**) 200 μm. Data are represented as mean **±** SEM (**C**, **D**, **G**). N = 10 ovaries per age group.

Interestingly, beyond wound healing capacity, there were prominent differences in the morphology of the OSE with age. The OSE in ovaries from reproductively young mice were typically flat and arranged in a single layer, whereas it appeared either multi-layered or columnar in reproductively old mice (Figure 13A, 13B). This was supported by the observation that the OSE was thicker in reproductively old compared to young mice; 15.2 ± 0.51 μm and 13.3 ± 0.42 μm, respectively (P = 0.007; Figure 13C). The OSE in reproductively old ovaries also displayed invaginations into the ovarian stroma, a phenotype that was unique to this age group. In fact, there were 9.9 ± 1.76 invaginations per ovarian section in reproductively old ovaries (P < 0.0001; Figure 13D). Moreover, the OSE of reproductively old mice contained regions where the OSE pulled away from the rest of the ovarian tissue, a phenomenon referred to as epithelial sheddings or outgrowths [49] (Figure 13E, 13F). There were 3.1 ± 0.92 outgrowths per aged ovarian section and only 0.2 ± 0.13 per young ovarian section (P = 0.0007; Figure 13G). These data provide multiple layers of evidence that OSE dynamics and properties are altered post-ovulation in mice of advanced reproductive age.

### Decreased COX2 expression may be a mechanism for ovulatory and wound healing dysfunction with age

**Figure 14:**
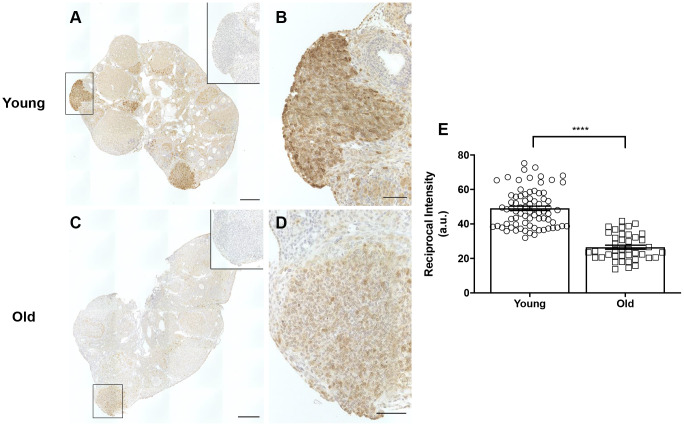
**COX2 expression is decreased in reproductively old ovaries.** (**A**) Representative image of a reproductively young ovary stained for COX2 expression. (**B**) Magnified image of boxed region in (**A**), a site of recent follicle rupture. (**C**) Representative image of a reproductively old ovary stained for COX2 expression. (**D**) Magnified image of boxed region in (**C**). The insets are IgG controls of the boxed regions. Scale bars are (**A**, **C**) 100 μm and (**B**, **D**) 25 μm. (**E**) Graph showing the reciprocal intensity values of recently ruptured follicles and formed CLs in tissue from reproductively young and old mice. A.u. stands for arbitrary units. A t-test was performed; asterisks indicate P < 0.0001. Data are represented as mean **±** SEM.

A potential mechanism that may contribute to impaired ovulation and post-ovulatory wound healing may be reduced cyclooxygenase 2 (COX2) activity. COX2 is an enzyme that converts arachidonic acid to prostaglandin H2 (PGH_2_), a committed step in prostaglandin synthesis [50]. Prostaglandins are involved in inflammatory processes including ovulation and help contribute to follicular rupture [51]. COX2 expression increases following the LH surge and is expressed in the granulosa cells of pre-ovulatory follicles [52]. COX2 also plays a role following ovulation, in that it is upregulated during the EMT, a process involved in wound repair that is induced in the OSE following ovulation [53]. Based on its broad roles prior to ovulation and during wound repair, we questioned whether there were age-associated differences in COX2 expression in the ovary. IHC on ovarian sections with an antibody against COX*2* showed significantly more COX2 expression in reproductively young mice than old, with expression particularly prevalent in areas of the ovary where follicle rupture recently occurred (Figure 14A–14D). Quantification of these areas confirmed that COX2 expression was significantly lower in reproductively old mice, which may contribute to impaired ovulation and post-ovulatory wound repair dysfunction (Figure 14E).

## DISCUSSION

In this study, we demonstrate that physiologic reproductive aging is associated with impaired ovulation, altered ECM status, reduced ovarian wound healing, and aberrant OSE morphology both *in vivo* and *in vitro*. These observations were made in a standard model of hyperstimulation and superovulation. It is important to note, however, that with age, animal weight increases, mimicking what also occurs in the human population as individuals reach middle age [22, 54]. Thus, it is possible that the hormone response to hyperstimulation and superovulation was variable, explaining the observed differences. This does not appear to be the case as serum estradiol and progesterone levels were similar between age groups. The similar response could be due to the supraphysiologic doses of hormones administered, but this is unlikely to be the case since similar phenotypes of reduced and failed ovulation with age were also observed when a lower dose of hormones was used. Although the hormone response was similar between age groups, there were interesting trends in the endocrinology which may become more pronounced with age. For example, serum estradiol tended to be higher in older animals. Large, hormonally responsive ovarian follicles respond to gonadotropins, undergo follicular growth, produce estradiol, and ovulate. Because there are fewer of these follicles in ovaries from older mice, we initially expected that the estradiol levels may be lower. However, adipose tissue is a prominent source of estrogen in postmenopausal women [55]. Since aged mice have increased adipose tissue relative to young, it is possible that increased estrogen from adipose tissue contributes to the observed elevation [22]. In contrast to estradiol, serum progesterone tended to be lower in older animals. This is consistent with what is observed in a human population, as progesterone tends to decline with increased weight and reproductive age [56, 57]. CLs are the primary source of progesterone, and we observed that CLs from reproductively old mice had a larger cross-sectional area and showed a greater degree of hypertrophy relative to controls [58]. Thus, these structural alterations in the CL may be a compensatory response to reduced functional output. It is important to note, however, that there was reduced CD31 expression and therefore, fewer endothelial cells in CLs from reproductively old mice. Because of these results, we cannot distinguish between the reduced number of nuclei in CLs from old mice being due to increased hypertrophy or fewer endothelial cells. However, the data considered in aggregate would suggest a luteal defect with age. It would be interesting to determine whether similar age-associated phenotypes are also observed in animals that are naturally cycling rather than hyperstimulated, but this is not a trivial study since animals of advanced reproductive age manifest irregular estrous cycles [59].

In our physiologic aging model, we clearly observed a decrease in the total number of ovulations as well as evidence of ovulatory dysfunction with age. Impaired ovulation occurs in genetic loss-of-function models and inhibition experiments targeting factors involved in different steps of the ovulatory process. For example, a complete failure of oocyte release occurs in mice lacking the progesterone receptor (PR), and ovulation is also blocked following knockdown of PR in monkey follicles, with both models exhibiting trapped oocytes in unruptured follicles more frequently than we observed in a model of physiologic aging [60, 61]. Ovulation requires dramatic changes in blood flow and angiogenesis, and placental growth factor (PGF), a member of the vascular endothelial growth factor (VEGF) family, is a critical regulator [62–64]. In primates, PGF antibody injection results in disorganized angiogenesis and trapped oocytes in addition to unruptured follicles with cumulus cell expansion, both phenotypes of failed ovulation consistent with our observations [65]. Additionally, proteolytic systems play a paramount role in ovulation because they are required for ECM degradation at the follicle apex, which allows for oocyte release. The matrix metalloproteinase (MMP) family of proteinases and the ADAM/ADAMTS family of metalloendopeptidases are critical for follicular rupture, and in animal models in which MMPs and ADAMTS are inhibited, the luteinized unruptured follicle phenotype occurs [66–68]. Additionally, ovulation is a highly inflammatory process during which large populations of leukocytes are recruited. It has been demonstrated that there are clearly distinct immune cells in ovaries from mice of advanced reproductive age, but the consequences of this in the context of ovulation are unknown [69]. It is possible that these molecules and cellular pathways are disrupted with age, and this is currently under investigation.

The age-associated defects in ovulation occur concurrently with an increase in ovarian collagen, and interestingly, this increase was also evident near and surrounding the CLs post-ovulation. Thus, the trapped oocyte and failed ovulatory phenotypes as well as the impaired wound healing ability we observed with age may in part be due to the physical constraints and rigidity imparted by increased collagen. This may have translational implications because collagen increases in the human ovary with advanced reproductive age [70]. Anecdotally, in the setting of Assisted Reproduction Technologies, the follicle walls are tougher in older women, making it more difficult to aspirate eggs at the time of egg retrievals in women of advanced reproductive age relative to younger individuals (personal communication, K. Lin).

Following ovulation, the ovarian wound must be repaired, which involves remodeling of the ECM and reformation of the OSE. We demonstrated that with advanced reproductive age, there is elevated collagen and decreased hyaluronan in ovaries post-ovulation. Additionally, there is less cell proliferation and apoptotic activity post-ovulation in ovaries from reproductively old mice. Proliferation and apoptosis both contribute to tissue wound healing and as such, this process appears impaired after wounding with age [40, 71]. Failure to properly remodel the ovary could result in accumulation of cellular debris, which may drive inflammatory pathways and contribute to fibrosis [72]. Interestingly, both fibrosis and inflammation increase in the somatic compartments of the ovary with age [15, 23].

We also observed aberrant OSE morphologies following ovulation in ovaries from aged mice, including a thick, multilayered OSE with columnar morphology, invaginations of the OSE into the ovarian stroma, and regions where the OSE pulled away from the rest of the ovary. These results are consistent with previous studies demonstrating similar phenotypes in ovaries from 22-month-old C57BL/6 mice and postmenopausal human ovaries [49, 73, 74]. One potential mechanism for these observed phenotypes may be an age-related increase in mammalian target of rapamycin (mTOR) activity, a conserved kinase that modulates aging [75]. Upregulation of mTOR activity is associated with prolonged acute injury in pulmonary epithelium and plays a role in the malignant transformation of ovarian cancer in humans [76, 77]. When 22-month-old mice exhibiting OSE invaginations and outgrowths were treated with rapamycin, an mTOR inhibitor, these pathological changes were suppressed [49]. Another potential mechanism responsible for these phenotypes may be dysregulation of the EMT. The EMT is a physiological process vital to tissue development during embryogenesis and in wound healing and tissue regeneration in adulthood [78]. During an EMT, epithelial cells lose their junctions and apical-basal polarity and acquire a migratory, invasive phenotype [79, 80]. The EMT is essential for wound healing, but if dysregulated, can lead to cancer development, scarring, and fibrosis in animal models and humans [81, 82]. Although multiple mechanisms are likely involved, the exact causes of impaired wound healing and aberrant OSE morphologies remain to be fully elucidated.

It is important to consider that we only examined animals of advanced reproductive age at one timepoint along the aging continuum (14-17 months old). To define the precise progression of ovulatory dysfunction with age, pursuing analysis of additional age groups would be of value. However, the phenotypes we observed are examples that are consistent with mild ovulatory dysfunction, which may have been obscured if younger mice were observed. Therefore, it is possible that we are seeing the beginning of ovulatory and remodeling dysfunction and that these phenotypes may be exacerbated at later ages. We also only examined a single timepoint post-ovulation, so future time course studies may be informative and are warranted. Nevertheless, these data provide an important platform to further consider ovulation and generalized wound healing in the context of advanced reproductive age. Although age-associated infertility is generally attributed to a decline in gamete quantity and quality, our results indicate that defects in ovulation may also contribute, which is especially relevant for natural conception.

Furthermore, these findings provide evidence for COX2 as a potential mechanism of impaired ovulation and post-ovulatory remodeling with age. COX2 is essential for ovulation which is impaired in estrogen sulfotransferase 1E1 (SULT1E1) knockout mice. SULT1E1 is an enzyme that sulfates estrogen and inactivates it. SULT1E1 knockout mice exhibit excessive estrogen, reduced COX2 expression and reduced ovulation [83]. COX2 inhibitors prevent follicular rupture in women and thus, COX2 is a potential contraceptive target [84]. The role of COX2 inhibitors as contraceptives supports the relationship between reduced COX2 expression and ovulation dysfunction with advanced reproductive age. Additionally, because COX2 is induced during the EMT and wound repair, reduced COX2 expression in ovaries from old mice, particularly in areas of recent follicle rupture suggest decreased COX2 as a potential mechanism involved in impaired OSE reformation.

Our findings also have broader implications beyond physiologic aging for other ovarian conditions characterized by increased fibrosis. For example, polycystic ovary syndrome (PCOS), the most common cause of anovulatory infertility in women of reproductive age, is in part characterized by elevated pro-fibrotic factors and ovulatory dysfunction [20, 85]. In addition, ovarian fibrosis is a late effect of both radiation and chemotherapy in the cancer setting [17–19]. Thus, ovulatory dysfunction may be predicted as an unintended consequence of these treatments. Our findings, therefore, have implications for further understanding the decline in fertility and ovarian function during aging and other conditions associated with fibrosis.

## MATERIALS AND METHODS

### Animals

Reproductively young CB6F1 mice (6-12 weeks old) were obtained from Envigo (Indianapolis, IN) and reproductively old CB6F1 mice (14-17 months old) were obtained from the National Institute on Aging Aged Rodent Colony (National Institutes of Health, Bethesda, MD). Mice were housed in a controlled barrier facility at Northwestern University’s Center for Comparative Medicine in Chicago under constant temperature, humidity, and light (14 hours light/10 hours dark). Mice were fed specific chows in their respective source facilities. Upon arrival to Northwestern University, mice were fed Teklad Global 2016 chow containing minimal phytoestrogens and no soybean or alfalfa meal (Envigo, Madison, WI) and given water *ad libitum*. All animal experiments described were approved by the Institutional Animal Care and Use Committee (Northwestern University) and performed in accordance with National Institutes of Health Guidelines.

### Superovulation, blood and organ harvest

Reproductively young and old mice received intraperitoneal (IP) injections of 5 IU pregnant mare serum gonadotropin (PMSG) (EMD Millipore, Danvers, MA) followed by 5 IU human chorionic gonadotropin (hCG) (Sigma-Aldrich, St. Louis, MO) 44-46 hours after PMSG injection (2.5 IU PMSG and hCG at same time points for half dose trial). 14-16 hours after the hCG injection, mice were weighed just prior to blood and organ harvest. After weights were taken, mice were anesthetized with 2-5% isoflurane and kept under this anesthesia for blood sample collection using venipuncture from the superior vena cava. In addition to 2-5% isoflurane, cervical dislocation was performed as a secondary method of euthanasia. Blood samples were allowed to clot for 90 minutes at room temperature. Afterward, samples were centrifuged for 15 minutes at 2000g (4615 rpm). Sera were separated into separate tubes, snap frozen, and stored at -20°C until use. Oviducts and ovaries were isolated and dissected out into dishes containing pre-warmed Leibovitz’s medium (L15) (Life Technologies Corporation, Grand Island, NY) containing 3 mg/mL polyvinylpyrrolidone (PVP) (Sigma-Aldrich) and 0.5% Pen Strep (Life Technologies) (L15/PVP/PS).

### Egg collection

Following oviduct and ovary isolation, the ampulla of each oviduct was noted for swelling. To collect cumulus oocyte complexes (COCs) containing mature eggs from each oviduct, ampullae were secured with forceps and nicked with a syringe needle to release the clutch of COCs ovulated. COCs were transferred to a drop of L15/PVP/PS media containing 1 μM hyaluronidase (Sigma-Aldrich) to facilitate cumulus cell separation. Cumulus cells were denuded from the COC by mechanical disruption to release eggs, and hyaluronidase was removed by rinsing eggs through large drops of L15/PVP/PS media. Eggs were assessed morphologically and scored as normal (defined by a clear plasma membrane and extruded polar body) or degenerate (dark, fragmented, granular, or shrunken cytoplasm). Transmitted light images were taken on an EVOS FL Auto Cell Imaging System (ThermoFisher Scientific, Waltham, MA) using a 40X objective.

### Hormone assays

Serum estradiol (E2) and progesterone (P4) concentrations were assessed by the University of Virginia’s Center for Research in Reproduction – Ligand Assay and Analysis Core. P4 was assessed using a commercially available kit for progesterone (mouse & rat) IBL ELISA according to manufacturer instructions (Immuno-Biological Laboratories Inc., MN, USA). The reportable range of the P4 assay was 0.15 - 40.00 ng/mL, and intraassay and interassay CVs were 4.18% and 5.74%, respectively. Where relevant, serum was diluted to 1:2 for samples that were beyond the detectable range and values beyond detectable range were extrapolated from the diluted results. E2 was assessed using a commercially available kit for estradiol (mouse and rat) ELISA according to manufacturer instructions (Calbiotech, CA, USA). The reportable range of the E2 assay was 3 – 300 pg/mL, and intraassay and interassay CVs were 6.90% and 6.45%, respectively.

### Histological processing and staining

Following the harvest protocol above, ovaries were placed in Modified Davidson’s solution (Electron Microscopy Services, Hatfield, PA) at room temperature for 2-4 hours with agitation, then overnight at 4°C. Samples were then washed three times, ten minutes each in 70% ethanol. Following this, samples were processed, dehydrated, and embedded in paraffin wax using an automated tissue processor (Leica Biosystems, Buffalo Grove, IL) per standard processing protocols. Embedded ovaries were sent to University of Kansas Medical Center (KUMC) for serial sectioning. Every fifth section of 5-μm-thick ovarian tissue was placed on slides for hematoxylin and eosin (H&E) staining and analysis. Tissues were stained following a standard H&E staining protocol, cleared with Citrisolv (Decon Laboratories Inc., King of Prussia, PA) in 3, 5-minute incubations, and mounted with Cytoseal XYL (ThermoFisher Scientific). For Picrosirius Red (PSR) staining, midsections of ovarian tissue were deparaffinized in Citrisolv, then rehydrated in a series of ethanol baths (100, 70, and 30%). Slides were stained with PSR solution consisting of Sirius Red (Direct Red 80, Sigma-Aldrich) in an aqueous saturated solution of picric acid (Ricca Chemical Company, Arlington, TX) at 0.1% w/v. Slides were incubated in PSR solution for 40 minutes at room temperature, then incubated in acidified water (0.05M hydrochloric acid) for 90 seconds. Tissue sections were then dehydrated in 100% ethanol (3, 30-second incubations), cleared in Citrisolv, and mounted with Cytoseal XYL. To quantify the area of ovarian tissue and CLs positive for PSR staining, scans of each ovarian section were taken at 20X using the EVOS FL Auto Cell Imaging System. ImageJ software (National Institutes of Health, Bethesda, MD) was used to quantify the area of PSR staining above a threshold set based on the staining in the mouse that appeared to have the most positive stain. This threshold was kept constant for all images analyzed for each mouse from both age groups.

### Analysis of corpora lutea number and characteristics

H&E stained ovarian sections were imaged using the EVOS FL Auto Cell Imaging System and CLs were identified in ovarian tissue. CLs stain lighter than other ovarian tissue, which allows them to be easily visualized with H&E staining. When a CL was seen for the first time in a section, it was marked and tracked in each subsequent section throughout which it appeared. Once a section was reached where it was no longer present, marking stopped and the total number of sections that the CL was present throughout was recorded. This was repeated for every CL. Failed ovulation events were characterized by two phenotypes: oocytes trapped within CLs and large antral follicles with cumulus cell expansion. The occurrence of these two phenotypes was recorded for each animal. Additionally, we analyzed the number of hemorrhaging CLs which we reported as the percent of hemorrhagic CLs per CL number. Corpora lutea size was quantified using the EVOS FL Auto Cell Imaging System. The perimeter of each CL was manually outlined and cross-sectional area measurements were calculated by the EVOS software. The number of ovarian sections that CLs appeared throughout was determined by the tracking process described above and the total number of histological sections was recorded. Nuclear: cytoplasmic ratio of CLs was measured by taking images of 5 CLs per ovary (or total CLs if an ovary contained fewer than 5) at 40X magnification. ImageJ was then used to count the number of nuclei present in the imaged areas.

### Immunohistochemistry

We performed IHC to detect Galectin-3 (ab53082; 1:1000 dilution; Abcam, Cambridge, UK) and Troma-1 (1:50 dilution; Developmental Studies Hybridoma Bank, Iowa City, IA), and COX2 (ab 15191; 1:1000 dilution; Abcam). Slides were deparaffinized in Citrisolv and rehydrated in graded ethanol washes (100, 95, 85, 70, and 50%). Antigen retrieval was performed by microwaving slides in 1X Reveal Decloaker (Biocare Medical, Pacheco, CA) at 50% power for two minutes followed by 10% power for seven minutes. Slides were washed with Tris-buffered saline (TBS) with 0.1% Tween-20 (TBST) twice for 15 minutes and then incubated in 3% hydrogen peroxide for 15 minutes at room temperature. Slides were rinsed in TBS and blocked using an avidin/biotin blocking kit (Vector Laboratories, Burlingame, CA). Slides were then rinsed in TBS and the area around the tissue was defined using a hydrophobic barrier pen (Vector Laboratories). Slides were incubated in block (10% normal goat serum in 3% BSA in TBS) for one hour. Block was removed and slides were incubated in the respective primary antibody diluted in 3% BSA in TBS overnight at 4°C. Slides were washed three times in TBST, 5 minutes each then incubated in secondary antibody, (biotinylated anti-rabbit for Galectin-3 and biotinylated anti-rat for Troma-1; 1:200) for 1 hour. Detection was performed using 3,3’-diaminobenzidine (DAB) using the DAB Peroxidase (HRP) Substrate Kit according to the manufacturer’s instructions (Vector Laboratories). The reaction was allowed to proceed for 30 seconds. Counterstaining was performed as follows: 40 second incubation in hematoxylin, 2 minutes running RO water, 20 seconds acidified ethanol, 1 minute running RO water, bluing reagent for 1 minute, and running RO water for 1 minute. Slides were dehydrated in ethanol baths (80, 95, and 100%), cleared in Citrisolv, and mounted with Cytoseal XYL. For Galectin-3, three slides of tissue per ovary were used, one from the beginning of the tissue sections, one from the middle, and one from the end of the tissue sections. The total number of Galectin-3 positive CLs were quantified and compared to the total number of CLs present for each ovary to determine the proportion of CLs in ovarian tissue that were regressing or from previous estrous cycles.

### Hyaluronan binding protein (HABP) assay

Tissue sections were deparaffinized in Citrisolv twice, 3 minutes each, and rehydrated in graded ethanol baths (100% for 2 minutes, 95% for 1 minute, and an additional 95% incubation for 2 minutes). Slides were rinsed in RO water for 1 minute and washed in 1X PBS on a rocker for 10 minutes. Endogenous avidin and biotin were blocked using an Avidin/Biotin blocking kit. Avidin was applied for 15 minutes, slides were rinsed in 1X PBS, then biotin was applied for 15 minutes. Incubations took place in a humid chamber at room temperature. Slides were rinsed in 1X PBS and incubated in normal goat serum for 20 minutes. Subsequently, control sections were incubated in 1 mg/mL hyaluronidase (Cat. #H3884-100MG; Sigma-Aldrich) in saline solution and experimental sections were incubated in saline alone for 1 hour at 37°C. Following a wash in 1X PBS, biotinylated HABP (Cat. #38599; Calbiochem, San Diego, CA) diluted in normal goat serum was added to all sections for a 1 hour incubation at room temperature. Slides were then washed in 1X PBS. Signal was amplified by incubating slides in ABC reagent (Vector Laboratories) for 30 minutes followed by TSA Plus Fluorescein System (Akoya Biosciences, Marlborough, MA). Both incubations took place in a humid chamber at room temperature. Samples were mounted in Vectashield HardSet Antifade Mounting Medium (Vector Laboratories) with DAPI to stain cell nuclei.

### Automated immunohistochemistry and quantitative microscopy

We performed automated IHC through the University of Washington Histology and Imaging Core (UW-HIC) with antibodies specific to CD31 (Clone SZ31, Dianova, Catalog Number DIA-310), E-Cadherin (Clone 24E10, Cell Signaling, Catalog Number 24E10), Ki67 (Clone D3B5, Cell Signaling, Catalog Number 12202), and cleaved caspase 3 (CC3, Clone D3E9, Cell Signaling, Catalog Number 9579) to assess OSE perimeter and thickness, proliferation, and apoptosis, respectively, in ovarian histologic sections. Utilizing the Leica Bond Rx Automated Immunostainer (Leica Microsystems), slides were first deparaffinized with Leica Dewax solution. Antigen retrieval was performed on all slides with EDTA, pH 9, at 100°C for 10 minutes for Ki67 and E-Cadherin stains and for 20 minutes for CD31 and CC3 stains. All subsequent steps were performed at room temperature. Initial blocking consisted of 10% normal goat serum (Jackson ImmunoResearch, Catalog Number 005-000-121) in tris-buffered saline for 20 minutes. Additional blocking occurred with Leica Bond Peroxide Block for 5 minutes. Slides were incubated with CD31 (1:250), E-Cadherin (1:400), Ki67 (1:400), or CC3 (1:250) primary antibodies in Leica Primary Antibody Diluent for 30 minutes. Next, a secondary antibody, goat anti-rabbit horseradish peroxidase polymerized antibody, was applied for 8 minutes. Antibody complexes were visualized using DAB detection 2X for 10 minutes. Tissues were counterstained with hematoxylin for 4 minutes followed by two rinses in water. Slides were removed from the automated stainer and dehydrated through graded alcohol to xylene. Dehydrated slides were coverslipped with synthetic mounting media. Unless otherwise specified all reagents were obtained from Leica Microsystems.

### Quantitative microscopy

Slides were scanned in brightfield with a 20X objective using the NanoZoomer Digital Pathology System (Hamamatsu City, Japan). The digital images were then imported into Visiopharm software (Hoersholm, Denmark) for analysis. All analysis was performed utilizing the Image Analysis Module within the software. For Troma-1 and E-Cadherin, regions of interest (ROI) were manually drawn around all ovarian tissue sections on each slide. The digital images of the Troma-1 slides were converted into grayscale values using two feature bands, Chromaticity Blue and H&E – Hematoxylin with a minimum 3 x 3 pixel filter. E-Cadherin digital images were also converted into grayscale values using two feature bands, HDAB – DAB and H&E – Hematoxylin with a median 11 x 11 pixel filter. Once converted, the software was trained to label positive, Troma-1 or E-Cadherin, and hematoxylin counterstain, based on a threshold of the feature band pixel values, creating a project-specific configuration. Images were processed in batch mode using this configuration to generate the ovarian perimeter, length of Troma-1 or E-Cadherin positive OSE, and ratio of the length of Troma-1 or E-Cadherin positive OSE per ovarian perimeter. For Ki67 slides, four ROIs were manually draw around follicles, corpora lutea, stroma, and the entire area of the ovary. Follicles were identified as containing an oocyte surrounded by layers of granulosa cells within a basement membrane. Corpora lutea were identified based on the morphology of luteinized cells, which have a characteristic hypertrophied appearance. The stromal area was defined as the total ovarian area minus the sum of the total follicular area and the total corpora lutea area. Once imported, the digital images were converted into grayscale values using two features, HDAB – HDAB and RGB – B. Visiopharm software was then trained to label Ki67 positive staining, and hematoxylin counterstain, based on a threshold of the feature band pixel values creating a project specific configuration. Images were processed in batch mode using this configuration to generate area positive Ki67, area tissue, total area, and a ratio of Ki67 per total area in the four regions outlined above.

For CD31-stained slides, ImageJ software (National Institutes of Health, Bethesda, MD) was used to manually draw ROIs around each CL of interest and to quantify the area of CD31 staining above a threshold set based on the staining in the CL that appeared to have the most positive stain. This threshold was kept constant for all CLs analyzed for each mouse from both age groups. The total area positive for CD31 stain was divided by the total CL area to determine the percentage of CL area that was CD31 positive. This was repeated for each CL in each tissue section. COX2 expression was quantified using reciprocal intensity, a method that measures chromogen intensity [86]. In this method, the maximum intensity value of a red-green-blue (RGB) color image in ImageJ is 250. For each CL and area of recent follicle rupture, an ROI was manually drawn and the intensity value was measured. This value was subtracted from 250, which gave a reciprocal intensity directly proportional to the amount of chromogen (DAB) present.

### Wound healing assay

Ovaries were isolated from reproductively young (9.5 weeks) and reproductively old (14-16 months) CB6F1 female mice primed with 5 IU PMSG and placed into L15/PVP/SP media. Ovaries were cut into six equal pieces which yielded 24 pieces for each age group. Each piece was encapsulated within a 0.5% alginate droplet and placed into 50 mM CaCl_2_ for 2 minutes to cross-link the alginate, which created a bead around the organoids. Encapsulated organoids were then placed into maintenance media and cultured for 0, 2, 4, 6, or 8 days (only day 0 and day 8 data shown). Maintenance media (MM) consisted of 30 μL α-MEM + GlutaMAX (ThermoFisher Scientific), 150 μL 0.5% Pen-Strep (Life Technologies), 300 μL 1% fetal bovine serum (FBS) (ThermoFisher Scientific), and 30 μL 0.1% insulin/transferrin/selenium (ITS) (ThermoFisher Scientific). Organoids were removed from MM on their respective days of culture and placed in alginate lyase solution (final concentration of 10 IU) for 30-35 minutes. Tissue pieces were then fixed in Modified Davidson’s solution or Ethanol-Formalin-Glacial acetic acid (EFG; 70%, 10%, 5% v/v, respectively) fixative at room temperature for 1 hour and then at 4°C overnight. The next day, they were washed 3 times with 70% ethanol, processed and embedded, and IHC was performed on sections with Troma-1 as described above.

### Statistical analysis

All results were graphed using GraphPad Prism Software Version 8.0.1 (La Jolla, California). Significant changes between groups were analyzed by students’ t-test and *P* values < 0.05 were considered statistically significant.

## Supplementary Material

Supplementary Figures
