# Supplementary material for: Ovulation and ovarian wound healing are impaired with advanced reproductive age
Source: Aging (Albany NY). 2020 May 14;12(10):9686–713. doi: 10.18632/aging.103237 (PMC7288922; doi:10.18632/aging.103237)
Supplement: Supplementary Figures [file aging-12-103237-s001..pdf]

SUPPLEMENTARY FIGURES

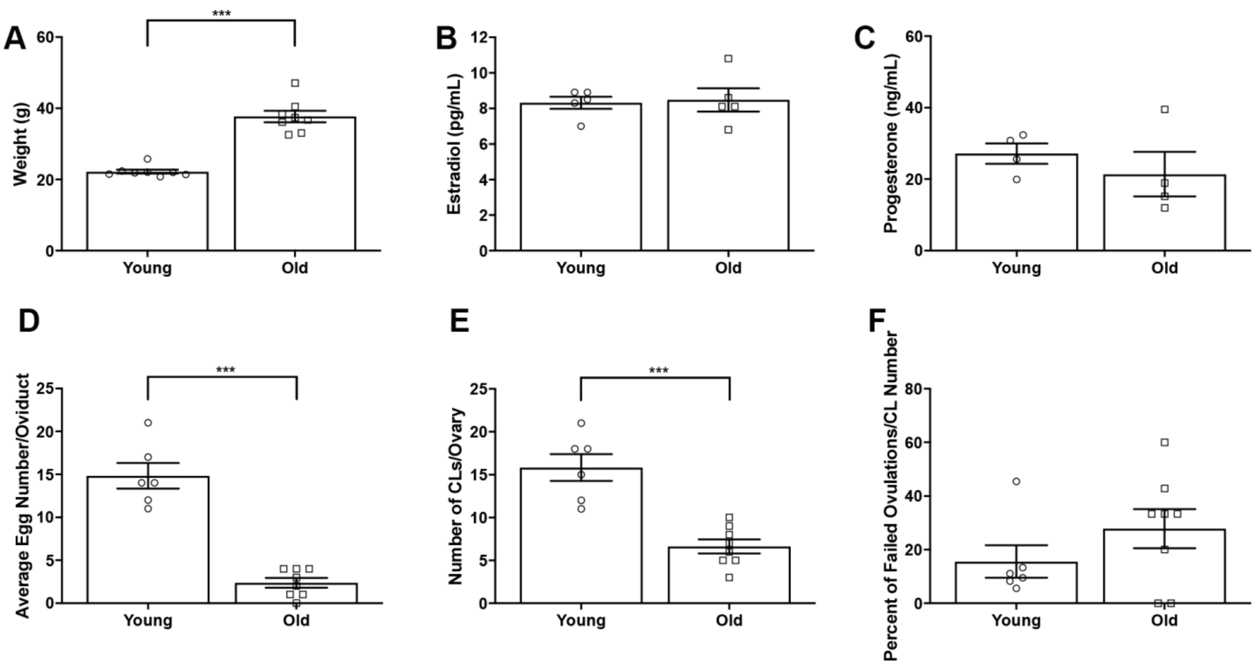

Supplementary Figure 1. Reducing the hyperstimulation and superovulation dose of PMSG and hCG (2.5 IU) also results in age-related failed ovulation phenotypes. (**A**) Weights of reproductively young and old mice (N = 8 mice per age group). A t-test was performed; asterisks denote significance (P = 0.0002). (**B**) Serum estradiol levels were assessed (reportable range 3–300pg/mL). A t-test was performed and there was no difference in estradiol levels between age cohorts. (**C**) Serum progesterone levels were assessed (reportable range 0.15–40.00 ng/mL). A t-test was performed and there was no difference between age cohorts. Graphs showing (**D**) the average number of eggs retrieved per oviduct and (**E**) the number of CLs tracked per ovary. T-tests were performed on both parameters; asterisks denote significance (**D**: P = 0.0003; **E**: P = 0.0007). (**F**) Graph showing the percent of failed ovulations per CL number for both age cohorts. A t-test was performed and the difference was not significant (P > 0.05). Data are represented as mean ± SEM.

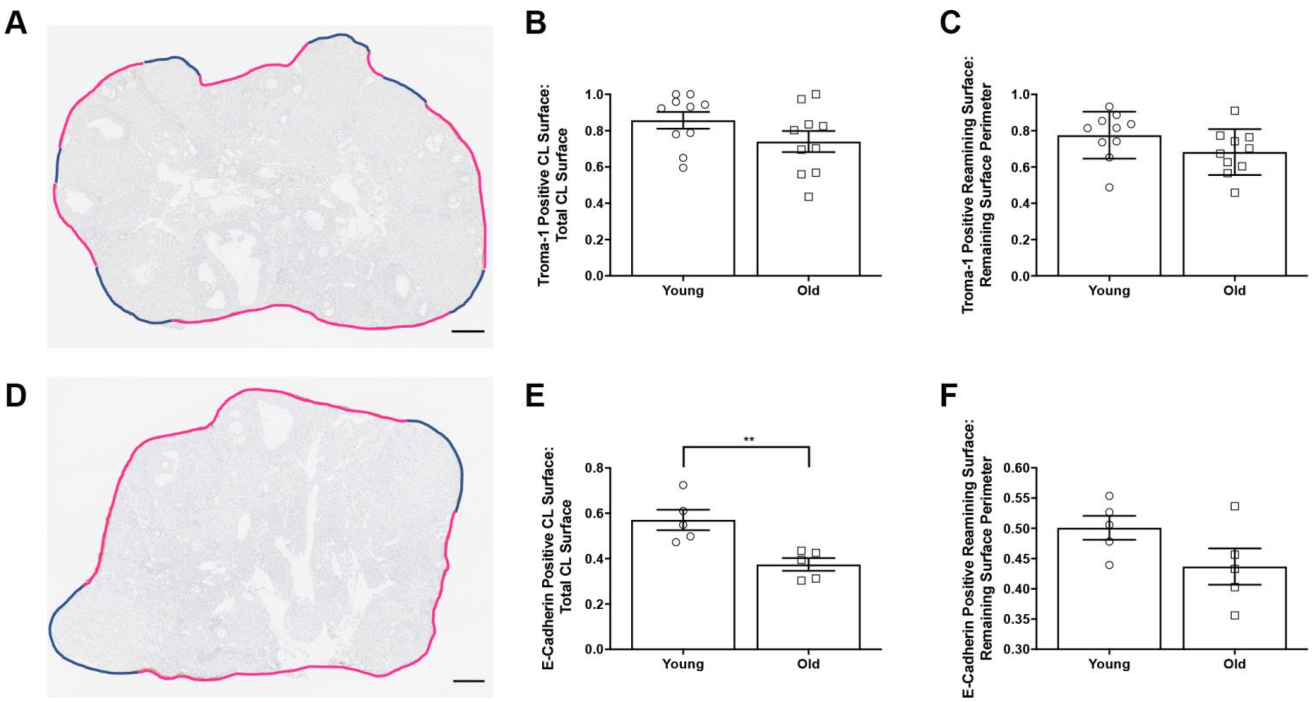

Supplementary Figure 2. Impaired remodeling of the OSE with advanced reproductive age tends to be specific to CLs. (**A**) Representative image of reproductively young ovary stained for Troma-1. CL surface is indicated by blue lines and remaining ovarian surface is indicated by pink lines. Graphs showing the proportion of the CL surface (**B**) and remaining ovarian surface (**C**) Troma-1 positive. (**D**) Representative image of reproductively old ovary stained for Troma-1. CL surface is indicated by blue lines and remaining ovarian surface is indicated by pink lines. Note that these images are the same as the tissue sections in Figure 11 (**A**, **B**). Graphs showing the proportion of the CL surface (**E**) and remaining ovarian surface (**F**) E-Cadherin positive.
